# ThermVision-DB: A synthetic LWIR thermal face dataset for privacy-preserving thermal vision research

**DOI:** 10.1016/j.dib.2026.112506

**Published:** 2026-01-28

**Authors:** Muhammad Ali Farooq, Waseem Shariff, Peter Corcoran

**Affiliations:** C3I Group, School of Engineering, University of Galway, Ireland

**Keywords:** Synthetic data generation, Diffusion models, Thermal imaging, Image-to-video translation, Privacy-preserving AI, Multimodal facial analysis, Computer vision benchmark

## Abstract

ThermVision-DB presents a synthetic long-wave infrared (LWIR) facial dataset designed to support research in privacy-preserving vision, thermal perception, and multimodal facial analysis. The dataset builds upon generative diffusion models to create photorealistic thermal facial images and video sequences capturing controlled variations in facial expression and head pose. Each synthetic identity is generated using text-to-image conditioning followed by video retargeting module, enabling precise control over pose angles, expression intensity, and frame-to-frame consistency. The dataset includes a diverse set of synthetic adult identities of both male and female genders with multiple facial expressions - such as neutral, smile, frown, and surprise and head-pose rotations spanning yaw, pitch, and roll. Data are provided in both image and video formats, accompanied by face localization annotations, landmark detections and identity labels. To ensure reusability and scalability, all samples are generated through a standardized pipeline using open-source models, allowing researchers to easily expand the dataset with additional synthetic identities while maintaining consistent thermal appearance and scene illumination. The synthetic generation process avoids the use of any personally identifiable visual data, ensuring compliance with FAIR and GDPR principles.

ThermVision-DB is intended for use in developing and benchmarking algorithms for facial detection, landmark localization, expression recognition, and head-pose estimation in the thermal domain. It also provides a foundation for research in synthetic-to-real transfer learning, privacy-safe biometric analysis, and cross-spectrum data fusion. The dataset is released for open research purposes under a non-commercial license, with full documentation and metadata available to facilitate reproducibility and integration with existing thermal vision benchmarks.

Specifications Table

**Table:** 

Subject	Computer Sciences
Specific subject area	*Generative AI, and Computer Vision.*
Type of data	Processed thermal frames and videos
Data collection	*Briefly describe how the data were collected. Please provide information on instruments you used (e.g., microscope, mass spectrometry, specific hardware or software etc., including relevant make/model details) as well as on methods used for collecting data or inclusion/exclusion criteria. You may also describe how the data were normalized. In case of questionnaires, please briefly describe the sources used to derive the question items. Max 600 characters (without spaces).* *Synthetic thermal facial data were generated by****tuning the FLUX.1-dev diffusion model****with text-to-image conditioning to produce diverse adult identities in the LWIR domain. For temporal synthesis, a****thermal domain–adapted image-to-video translation pipeline****based on****LivePortrait****was employed to render expression and head-pose variations. All outputs were normalized for thermal intensity and pose alignment.*
Data source location	*Please mention where the data were collected (e.g. geographical coordinates) or where the data are stored (typically your affiliation).* *Laboratory: C3Imaging, University of Galway* *Institution: School of Engineering, University of Galway* *City/Town/Region: Galway Country, Ireland*
Data accessibility	***Please note:****All raw data referred to in this article must be made publicly available in a data repository prior to publication. Please indicate here where your data are hosted (the URL must be working at the time of submission and editors and reviewers must have anonymous access to the repository):* Repository name: MAli-Farooq/ThermVision-DB · Datasets at Hugging Face [1] Data identification number: 10.57967/hf/7026 Direct URL to data: https://huggingface.co/datasets/MAli-Farooq/ThermVision-DB The dataset is publicly available on **Hugging Face** and the project repository, with documentation and resources accessible via https://mali-farooq.github.io/ThermVision. **Repository name:***MAli-Farooq/ThermVision-DB · Datasets at Hugging Face* **Data identification number:** DOI: https://doi.org/10.57967/hf/7026 **Direct URL to data:** MAli-Farooq/ThermVision-DB · Datasets at Hugging Face [1] Project Website: https:mali-farooq.github.io/ThermVision [2] Code: https://github.com/mali-farooq/ThermVision **Instructions for accessing these data:** All dataset files, metadata, and documentation are freely available for research and non-commercial use. Users can access or download the data directly from the Hugging Face repository without login requirements. Detailed dataset structure and usage examples are provided in the accompanying README.md and project website https://mali-farooq.github.io/ThermVision.
Related research article	If your manuscript supports a related research article, please cite this article here. If your manuscript is not related to a research article, please state ‘none’. You should **list only one article here**. Please upload a copy of your related research article to your submission.
	***Related research article:*** Farooq, M. A., Shariff, W., & Corcoran, P. (2025). ThermVision: Exploring FLUX for Synthesizing Hyper-Realistic Thermal Face Data and Animations via Image to Video Translation. In Proceedings of the ACM International Conference on Multimedia (ACM MM 2025), Dublin, Ireland. 10.1145/3746027.3755448 [3]

## 1 Value of the Data

• **High-Quality Synthetic Thermal Data:** This data consists of thermal face images generated via diffusion models, and image-to-video translation methods simulating realistic facial and thermal variations. They provide a valuable resource for developing and evaluating algorithms in contexts where real thermal data are scarce or privacy sensitive.
• **Supports GDPR Compliance and Ethical Research:** Since the data are fully synthetic, they avoid issues related to personal data collection, consent, and GDPR restrictions. Researchers can freely use the dataset without handling sensitive personal information.
• **Facilitates Reproducibility and Benchmarking:** Publicly available synthetic thermal data allow other researchers to replicate experiments, validate model performance, and benchmark algorithms consistently without relying on restricted or costly real datasets.
• **Enables Multi-Modal and Cross-Domain Studies:** The dataset can be combined with other modalities (e.g., RGB, depth, event-based sensors) to explore multi-modal learning, domain adaptation, or cross-spectral recognition tasks.
• **Encourages Methodological Innovation:** Researchers can use these synthetic images to test new preprocessing, augmentation, and model training strategies, including diffusion-based approaches, while mitigating privacy risks.

## 2 Background

The dataset was compiled to address the scarcity of high-quality thermal face imagery in the LWIR (long-wave infrared) modality, suitable for research in privacy-sensitive, low-light, or zero-light conditions. It is based on diffusion-based generative modelling, specifically latent diffusion architectures, to synthesize realistic LWIR thermal face images with controlled variations in pose, expression, and thermal appearance. The dataset, named ‘ThermVision-DB’, contains 30,200 frames across 100 videos at 512 × 512 resolution, covering 50 unique subjects with the potential to generate unlimited variations using open-sources thermally tuned Flux text-to-image diffusion models. Annotations include face bounding boxes, facial landmarks, identity labels, and gender, along with attributes such as head poses, facial expressions, hairstyles, and accessories, ensuring diversity and gender parity. Further, 2D facial subjects - including male and female identities with diverse facial hairstyles, facial accessories and attributes - were synthesized using text prompts to control variations in head pose, expression, and thermal appearance.

This dataset complements the related research article by providing a curated, reusable synthetic corpus for algorithm development, benchmarking, and replication studies. Researchers can employ ThermVision-DB for tasks including LWIR thermal face detection, recognition, and multi-modal fusion, without the need of acquiring sensitive personal data. By providing structured annotations and diverse attributes, it enables systematic evaluation and experimentation under varied LWIR thermal imaging conditions.

## 3 Data Description

*This section describes your dataset. Refer to all the data folders, subfolders, and files in the repository individually, irrespective of whether they relate to raw or analyzed data, and make sure that the reader can follow the structure of your dataset.*

*Please use visual aids (such as tables, graphs, or figures with captions) to familiarize the reader with your dataset, but* ***do not offer background, interpretations, or conclusions****.*

**ThermVision-DB** is a comprehensive synthetic thermal facial dataset designed to support research in thermal imaging, biometrics, multimodal perception, and privacy-preserving model development. The dataset includes both **video sequences** and **2D facial images**, each generated using advanced diffusion and image-to-video synthesis techniques to simulate realistic thermal characteristics, facial dynamics, and demographic variations.

### 3.1 ThermVision-DB video data

As mentioned in Table 1, the video component of ThermVision-DB contains 100 thermal videos representing 50 synthetic subjects, with an equal distribution of 25 males and 25 females. Each subject has two distinct video sequences, providing a combined total of 604 frames per subject. In total, the video dataset includes 30.2K frames in MP4 format at a resolution of 512 × 512. Each video is accompanied by annotations such as identity labels, gender, and face bounding boxes. The sequences also include diverse visual characteristics - including head pose variations, facial expressions, hairstyles, and accessories - ensuring a rich and realistic set of conditions for tasks like thermal face recognition, tracking, expression analysis, and temporal modelling.

**Table 1:** Overview of the **ThermVision-DB Video Dataset**, including subject distribution, video specifications, total frame count, annotations, and key facial attributes represented in the thermal video sequences.

Total Subject/ Gender	Video Format and Image Resolution	Per Subject Data	Total Dataset Size	Annotations	Attributes
50 (can generate unlimited) 25 male, 25 female	MP4, 512 × 512	2 distinct video sets, totalling 604 frames.	30.2K frames, 100 videos (2 videos each subject)	Gender, face boxes, Identity	Head poses, expressions, hairstyles, facial accessories, gender parity

### 3.2 ThermVision-DB 2D facial image data

In addition to videos, ThermVision-DB offers a 2D thermal facial image dataset, also at 512 × 512 resolution. As mentioned in Table 2, the images span multiple categories designed to capture a broad range of conditions, including frontal faces, head-pose variations, facial accessories such as glasses and masks, male subjects with beards, and a mixed category that combines multiple facial variations. Both male and female subjects are represented across these categories, providing balanced demographic coverage and enabling robust evaluation of cross-domain and pose-invariant thermal imaging models.

**Table 2:** Summary of the **ThermVision-DB 2D Facial Image Dataset**, detailing image categories, content descriptions, resolution, and gender distribution across various thermal facial variations.

Category	Description / Content Type	Image Resolution	Gender
Frontal Pose	Frontal-facing thermal face images of male and female subjects	512 × 512	Male / Female
Facial Accessories	Images including glasses, and masks		Male / Female
Beard	Thermal facial images featuring subjects with beards.		Male
Mix	Mixed data combining multiple poses and facial variations.		Male / Female
Head poses	Thermal face images with varying head poses and orientations.		Male / Female

### 3.3 Dataset size, Accessibility, and Reproducibility

From a practical standpoint, distributing a static dataset containing hundreds or thousands of identities would substantially increase storage and bandwidth requirements. The current release already comprises approximately 21 GB of data [1,2], including identity-wise samples, pose variations, cropped face images, and video sequences. Scaling this volume linearly would result in datasets of several tens or even hundreds of gigabytes, thereby creating accessibility barriers for many researchers. By prioritizing generative scalability over static data volume, as further discussed in the subsequent sections, ThermVision-DB offers a more flexible, sustainable, and reproducible solution for large-scale thermal face research.

### 3.4 Generative scalability and identity expansion

While the current public release of ThermVision-DB contains 50 unique synthetic identities, this number should not be interpreted as a limitation of the proposed approach. Instead, ThermVision-DB is designed as a scalable (generative framework), rather than a fixed-size dataset.

2D Thermal Facial Frame Synthesis: The core contribution of this work is the release of a fine-tuned FLUX diffusion model along with dual clip loader models, FLUX.1 Dev UNet model, and VAE model files for LWIR thermal facial synthesis. Once fine-tuned, the diffusion model enables the generation of an effectively unlimited number of identity-distinct thermal faces by sampling new latent representations, without requiring additional data collection or retraining. Identity diversity is achieved through stochastic latent sampling combined with controlled prompt conditioning, allowing users to generate new male or female identities with varied facial structure, thermal appearance, expressions, and head poses on demand.

The current subset of 50 identities was selected as a representative sample to

(i) Demonstrate identity diversity and thermal realism.
(ii) Validate downstream tasks such as face detection, landmark estimation, and expression analysis.
(iii) Maintain a manageable dataset size to support accessibility, reproducibility, and public dissemination. Importantly, increasing the number of identities is a user-driven process that can be performed locally, enabling researchers to scale the dataset to hundreds or thousands of identities tailored to their specific experimental needs.

**Fig. 1:**
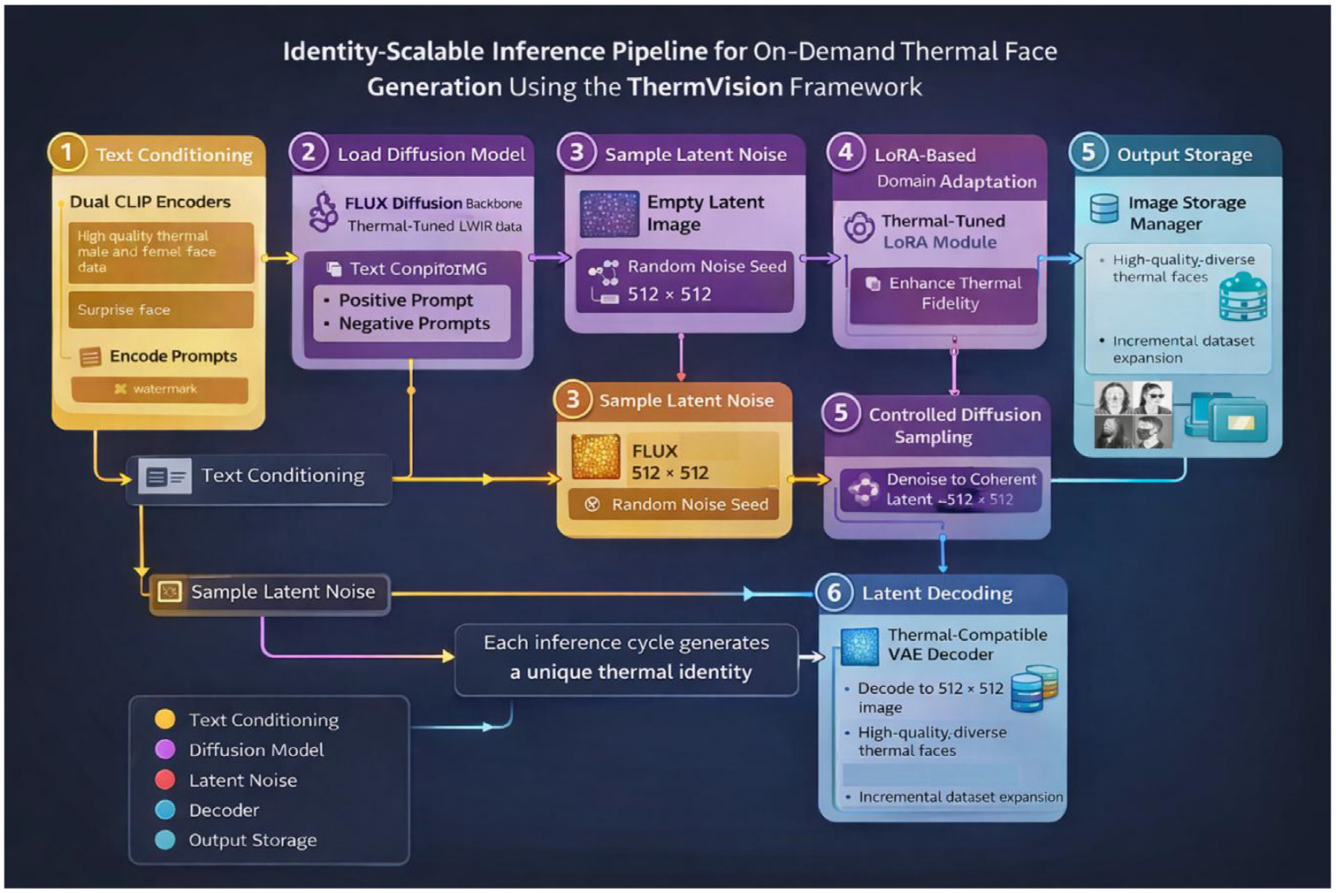
Illustrative overview of the ThermVision identity-scalable inference pipeline. Text prompts are encoded using dual CLIP encoders, while identity diversity is introduced through stochastic latent noise sampling. A FLUX diffusion model fine-tuned on LWIR facial data, enhanced with a thermal LoRA module, generates coherent thermal facial representations that are decoded into 512 × 512 thermal images. By resampling latent noise and prompts, the pipeline enables on-demand generation of hundreds or thousands of unique synthetic thermal facial identities.

To explicitly illustrate this scalability, Fig. 1 illustrates the inference pipeline used to generate synthetic LWIR thermal facial identities on demand. The pipeline is designed to enable scalable identity generation by sampling new latent representations without retraining the model. Each inference pass produces a distinct synthetic identity, controlled through prompt conditioning and latent noise initialization. Table 3 summarizes step by step processes illustrated in Fig. 1 to generate scalable thermal synthetic identities. Furthermore, the complete JSON workflow for generating additional thermal facial identities using the ComfyUI tool is publicly available on our GitHub repository [2].

**Table 3:** Inference workflow for identity-scalable thermal face generation

Steps	Description
1. Text Conditioning via Dual CLIP Encoders	Text Conditioning via Dual CLIP Encoders: Semantic control is provided using a dual CLIP-based text encoding mechanism. Positive prompts specify desired facial and demographic attributes (e.g., thermal facial appearance, gender, expression), while negative prompts suppress undesired artifacts such as watermarks or unrealistic textures. This dual conditioning ensures semantic consistency and controllability across generated identities.
2. Thermal-Base Diffusion Model Loading	A FLUX diffusion backbone is loaded. This model captures thermal-specific characteristics, including heat distribution patterns, sensor noise behavior, and low-frequency facial structure
3. Identity Variation via Latent Noise Sampling	An empty latent tensor is initialized at a fixed spatial resolution. New identities are generated by sampling different random noise seeds in the latent space. Each seed corresponds to a unique synthetic identity, enabling scalable identity generation without additional data collection or retraining.
4. LoRA-Based Domain Adaptation	A thermally tuned Low-Rank Adaptation (LoRA) module is applied during inference to refine identity realism and thermal consistency. The LoRA selectively modulates the diffusion process to preserve facial structure while enhancing domain-specific thermal features.
5. Controlled Diffusion Sampling	The conditioned latent representation is passed through a diffusion sampler with a fixed guidance scale and step range. This process progressively denoises the latent space into a coherent thermal facial representation while maintaining identity diversity across different samples.
6. Latent Decoding to Image Space	The final latent representation is decoded using a thermal-compatible variational autoencoder (VAE) to produce a 2D thermal facial image at 512 × 512 resolution.
7. Output Storage and Dataset Expansion	Generated images are stored locally. By iterating this pipeline with different noise seeds and prompt variations, hundreds or thousands of unique thermal facial identities can be generated on demand, enabling scalable dataset expansion.

Reproducible Image-to-Video Retargeting for Thermal Facial Animation: To ensure full reproducibility of the proposed thermal facial animation pipeline, all fine-tuned model components used in this work are publicly released via our Hugging Face repository. These models can be downloaded and stored locally following the directory structure shown below, enabling researchers to reproduce both the static thermal face synthesis and the subsequent animation stages.



Using the released Flux diffusion models and the provided ComfyUI inference workflow, we can locally generate identity-distinct LWIR thermal facial images as single 2D frames. Once a synthetic thermal face is generated, it can be used as the target identity for facial animation. The static thermal face image is imported into the LivePortrait framework [4], which supports image-to-video retargeting using a separate driving video as input. From the driving video, facial expressions, lip movements, and head pose dynamics are extracted and transferred to the thermal identity, producing an identity-consistent thermal facial animation sequence.

In this modular setup, the diffusion-based generative model and the video retargeting framework operate independently but complementarily. The fine-tuned FLUX diffusion models enable scalable synthesis of identity-diverse thermal faces, while LivePortrait performs temporal motion transfer without requiring identity-specific thermal videos or retraining. By combining these two components, the complete pipeline supports reproducible generation of both static LWIR thermal identities and dynamic thermal facial animations, as demonstrated in this work.

## 4 Experimental Design, Materials and Methods

The ThermVision-DB dataset was generated using a two-stage diffusion-based framework designed for synthetic LWIR (long-wave infrared) face data generation and retargeted animation. The framework comprises two primary stages: (S1) Thermal Image Generation using Text based Conditioning and (S2) Image-to-Video Translation using Video Retargeting Module.

***Stage 1: Thermal Image Generation using Text to Image Conditioning***

**Fig. 2:**
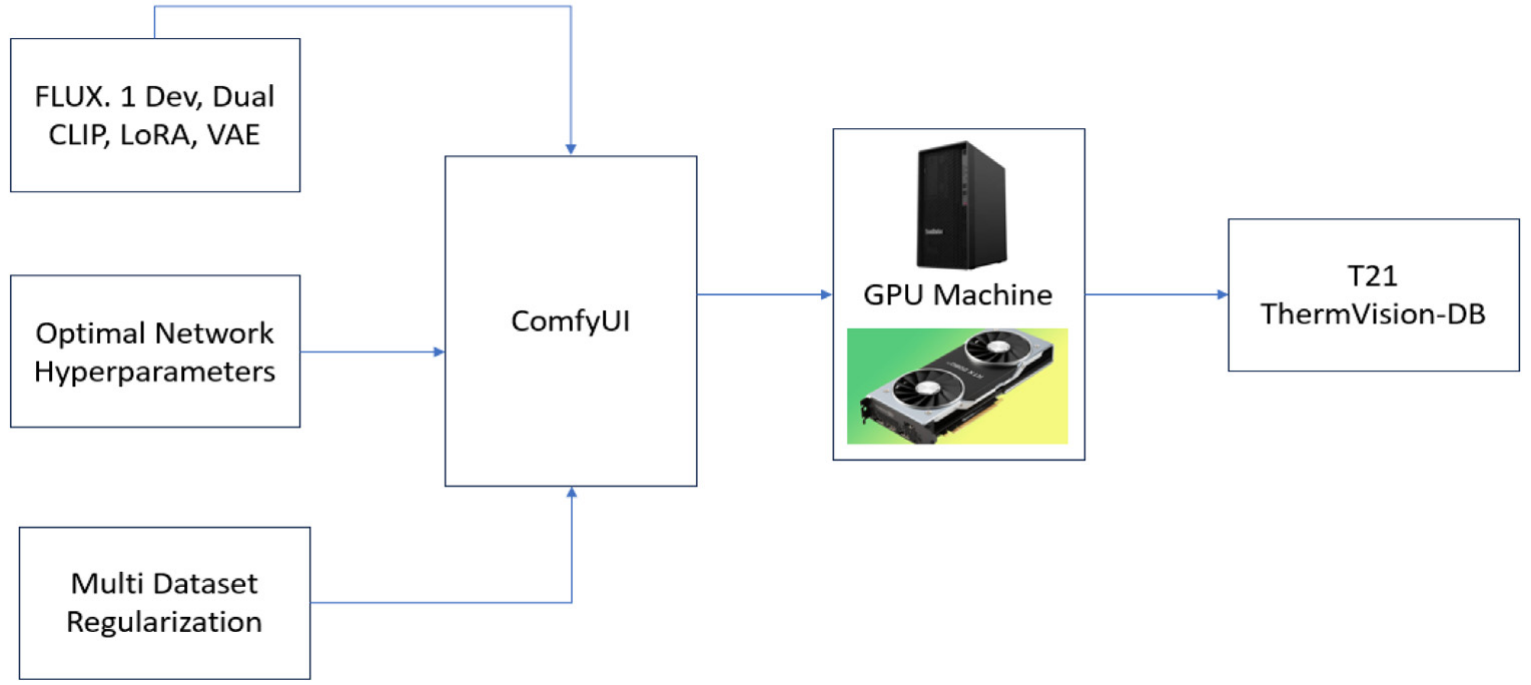
FLUX Training Setup: The setup was implemented using ComfyUI, with all optimal diffusion and LoRA fine-tuning parameters configured through a modular node-based pipeline to achieve controlled generation and thermal consistency.

In the first stage, both male and female thermal datasets were used to train text-to-image (T2I) diffusion models, including FLUX.1 DEV [5], LoRA [6], VAE, and CLIP [7]. The framework was implemented in ComfyUI, enabling modular workflow orchestration, model chaining, and parameter optimization as shown in Fig. 2.

**Fig. 3:**
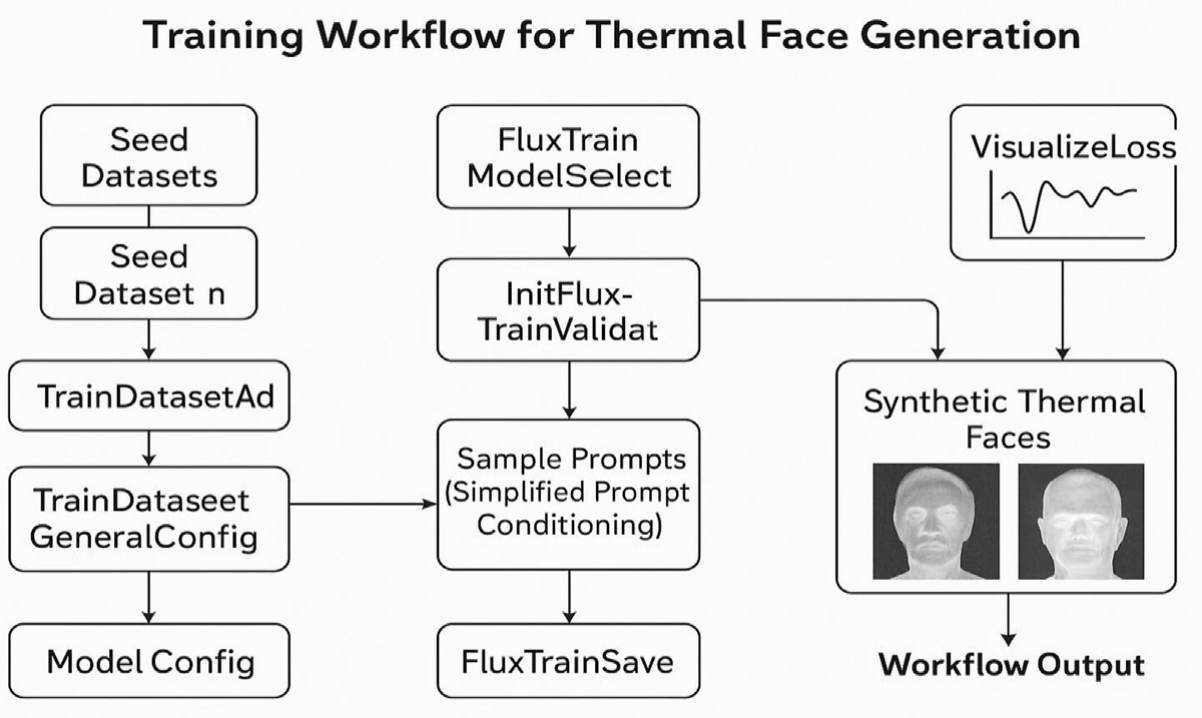
FLUX Training Workflow for generating 2D thermal LWIR frames by employing UNET, FLUX LoRA, CLIP and VAE.

Fig. 3 illustrates the *ComfyUI-based training pipeline* designed for fine-tuning the Flux LoRA model to generate synthetic LWIR (thermal) facial images using multiple seed datasets. The workflow is structured as a sequential modular process consisting of five major stages - dataset input, configuration, iterative training, validation, and output generation - implemented through ComfyUI’s node-based visual interface.

**1. Seed Dataset Input and Preparation**

The workflow begins with multiple seed datasets (n = 4) - Tufts Face Database [8], CARL Dataset [9], Charlotte-ThermalFace [10], and a locally acquired LWIR dataset [11].

Each dataset is added via TrainDatasetAdd nodes at varying resolutions (512 × 512, 768 × 768, and 1024 × 1024) to introduce scale and diversity in training.

The TrainDatasetGeneralConfig node manages dataset indexing, text-prompt pairing, and thermal-specific augmentations such as temperature-based intensity normalization.

As mentioned for model adaptation and domain tuning, four different thermal datasets were employed among which three are obtained from open-source public repository and fourth dataset is acquired locally. These datasets introduced broad diversity in facial pose, accessories, and gender balance, enabling a more generalized and precise thermal generative model.

*Public Thermal Dataset Acquisition*

For the Tufts Thermal Face Dataset, data were acquired using a FLIR Vue Pro LWIR camera, with participants seated in close proximity to the camera against a uniform blue background. The camera was mounted on a tripod, and its height was manually adjusted to align the subject’s face with the image center. The camera–subject distance was strictly controlled throughout the acquisition process. Although thermal imaging does not rely on visible illumination, constant and diffused ambient lighting was maintained to ensure stable recording conditions and subject comfort.

The CARL Dataset [9] was captured using a TESTO 880-3 thermographic camera equipped with an uncooled detector operating in the 8–14 ®m spectral range. Subjects were positioned at a fixed distance of 135 cm from the camera, with all tripods and support structures placed on predefined ground markings to ensure consistent acquisition geometry. A pair of halogen light sources was positioned approximately 30° off the frontal direction and at a distance of about 3 m from the subject, matching the room’s artificial lighting conditions and maintaining stable ambient temperature.

For the Charlotte-ThermalFace Dataset, data were recorded across four different ambient temperatures, with air temperature deliberately varied from 20.5°C (69°F) to 26.5°C (80°F). Images were acquired at multiple camera–subject distances, ranging from 1 m to 6.6 **m**, and across **25** distinct head positions, enabling systematic analysis of thermal appearance variations due to distance, pose, and environmental temperature.

**Fig. 4:**
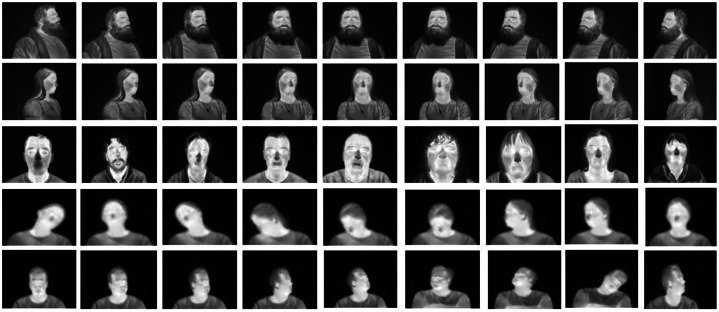
Seed (training) data samples acquired from three different publicly available thermal datasets including Tufts Face Database [8], CARL Dataset [9], Charlotte-ThermalFace [10].

Fig. 4 shows the seed data samples from public datasets covering different facial characteristics, head poses, genders, and attributes.

*Local Thermal LWIR Dataset Acquisition:*

**Fig. 5:**
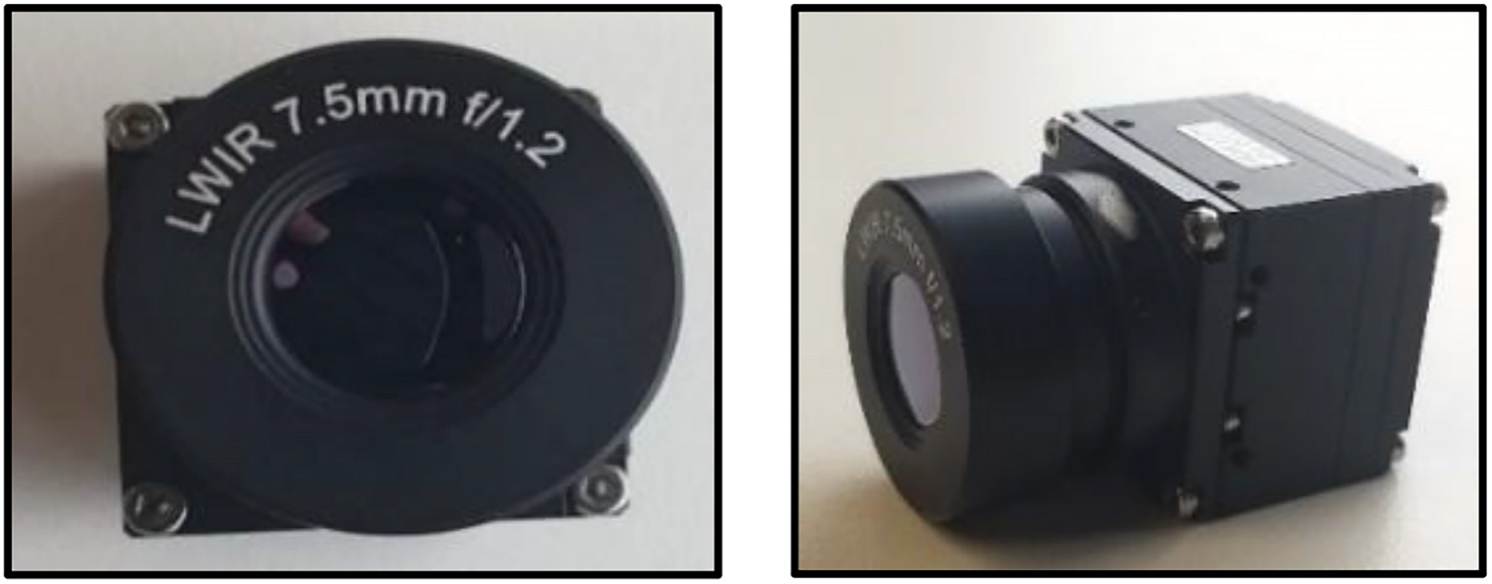
Thermal LWIR camera sensor used for acquiring data locally in lab environment.

The local thermal data was captured using a 640 × 480 uncooled LWIR camera as shown in Fig. 5 equipped with a 7.5 mm lens and an f/1.2 aperture, providing high sensitivity and sharp contrast in the 8–14 µm wavelength band.

To take comprehensive facial information during the data acquisition process, we have calculated other important parameters including the lens aperture, angular Field of View (AFOV), height and width of the sensor, and working distance as shown below.

(1) $$F-Number=\;\frac{Focal\;Length\;\left(f\right)}{Diameter\;\left(D\right)}$$

(2) $$Diameter\;\left(D\right)=\;\frac{Focal\;Length\;\left(f\right)}{F\;Number\;}=\;\frac{7.5}{1.2}=6.25\;\approx \;6\;mm$$

(3) HeightofSensor(h)=HorizontalPixels*PixelsPitch=640*17=10.88mm

(4) WidthofSensor(w)=VetriclePixels*PixelsPitch=480*17um=8.16mm

(5) $$AFOV\;=\;2*\;\tan^{-1}\frac{h}{2f}\;=\;2*\;\tan^{-1}\frac{10.88\;mm}{2*\;7.5\;mm}\;=71.9\;\approx 72\;Deg$$

(6) $$Working\;Distance\;\left(WD\right)=\;\frac{Focal\;Length\;\left(f\right)*\;HFOV}{height\;of\;Sensor\;\left(h\right)}=\;\frac{7.5\;*890}{10.88\;}\approx 60\;cm$$

**Fig. 6:**
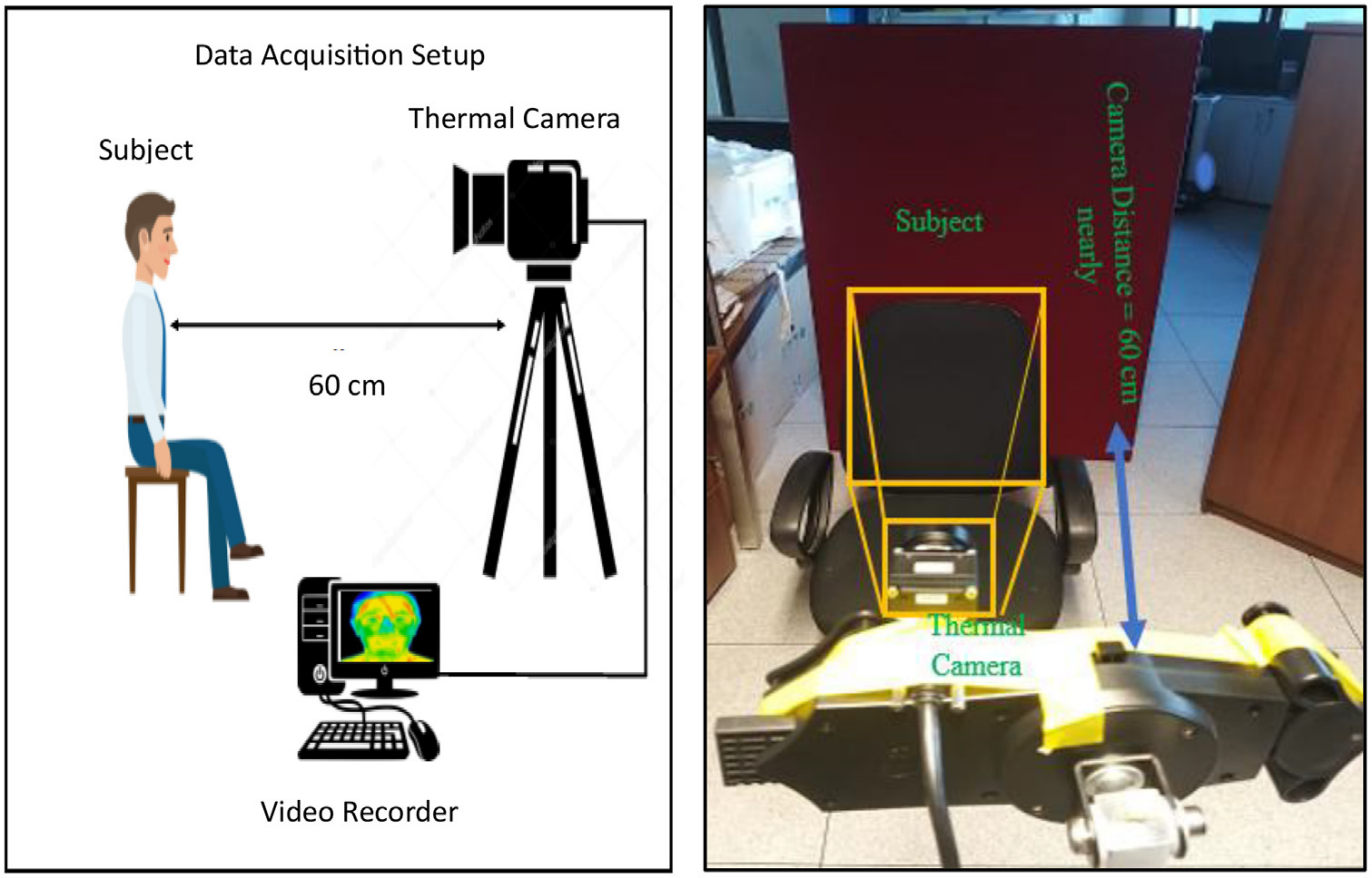
Indoor lab environment data acquisition setup

**Fig. 7:**
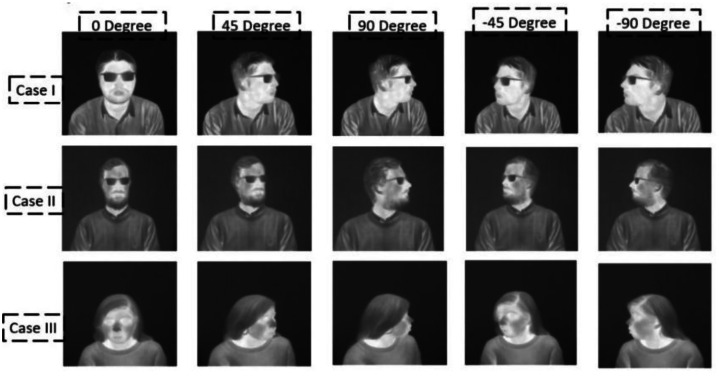
Locally acquired thermal face data samples [11] of male and female subject covering various facial angles and with facial accessories.

The data is collected by mounting a camera on a tripod at a fixed distance of 60-65 cm. The height of the camera is adjusted manually to align the subject’s face centrally in the field of view. Shutterless camera calibration at 30 FPS is used to acquire the data. All recordings were conducted in a controlled indoor environment under stable ambient conditions, with room temperature maintained within a typical indoor range and without direct external heat sources influencing the subject. As the sensor operates in the long-wave infrared (LWIR) spectrum, no visible-light illumination was required; instead, the recorded thermal signatures are solely governed by the subject’s natural facial heat distribution and the surrounding ambient temperature. The data acquisition setup is shown in Fig. 6. The data was gathered by recording videos stream of each subject covering different facial poses and then generating image sequences from the acquired videos. Fig. 7 shows the image samples from locally acquired uncooled LWIR thermal camera.

The primary objective of incorporating all these datasets was to capture a wide range of variations in facial poses, accessories, and gender balance (male and female), enabling the development of a more precise and generalizable text-to-image diffusion model.

**2. Model Configuration and Hyperparameter Setup**

The Flux1-dev model [5] is initialized through the FluxTrainModelSelect node, loading pretrained weights (safetensors) along with associated VAE, CLIP [7], and T5 text encoders.

Table 4 summarizes the complete training configuration used for the Flux-based diffusion model within the ThermVision framework. The model was trained using a learning rate of 0.0004, a network dimension of 64, and 3,000 training steps.

**Table 4:** Flux model training and optimization parameters.

Parameter	Value	Description
Network_dimension	64	Base network feature dimension
Network_alpha	64.00	Channel scaling factor for stable learning
earning_rate	0.0004	Fixed learning rate applied throughout training
learning_scheduler	Constant	Constant scheduler to maintain steady learning rate
optimizer_type	CAME	Confidence-guided Adaptive Memory Efficient Optimizer
max_train_steps	3000	Total number of training iterations
apply_t5_attn_mask	true	Enables T5 attention mask during text conditioning
cache_latents	disk	Stores latent representations on disk for efficiency
cache_text_encoder_outputs	disk	Caches encoded text embeddings on disk
blocks_to_swap	1	Number of network blocks swapped to manage memory load
weighting_scheme	logit_normal	Balances sample weighting during diffusion
logit_mean	0.00	Mean of the logit normalization distribution
logit_std	1.00	Standard deviation for logit normalization
mode_scale	1.29	Scaling factor for diffusion stability
timestep_sampling	shift	Employs shifted timestep sampling for smoother training
sigmoid_scale	1.0	Sigmoid scaling coefficient
model_prediction_type	raw	Raw output prediction type for diffusion
CFG_scale (Train and Val)	3.00	Classifier-Free Guidance scale for balancing realism and diversity
discrete_flow_shift	3.1582	Discrete latent flow offset
highvram	false	Memory optimization for GPUs
fp8_base	true	Enables FP8 base precision for performance
gradient_dtype	bf16	Gradient precision type
save_dtype	bf16	Model checkpoint precision type
attention_mode	sdpa	Uses Scaled Dot-Product Attention for efficiency

To optimize both performance and stability, a Confidence-guided Adaptive Memory Efficient (CAME) optimizer was employed along with a constant learning rate scheduler. These methods ensured stable convergence under limited GPU memory conditions.

Additional strategies such as logit_normal weighting, shift timestep sampling, and scaled dot-product attention (SDPA) were applied to maintain balanced gradient propagation and enhance diffusion consistency. Caching mechanisms were used for both latent and text encoder outputs to reduce runtime memory usage. The model utilized bfloat16 (bf16) precision for gradient updates and checkpoint storage to improve computation efficiency.

A Classifier-Free Guidance (CFG) scale of 3.0 was consistently applied during both training and validation phases to balance realism and diversity in the generated thermal imagery.

These combined configurations enabled the generation of high-fidelity, text-conditioned synthetic LWIR thermal images that exhibit stable facial identity, pose, and illumination characteristics.

**3. Training Loop and Sample Generation**

The workflow is divided into four iterative training loops, each performing fine-tuning over batched samples. Simplified text conditioning is applied through dual CLIP loaders using both *positive* and *negative* prompts for identity and expression control. Intermediate synthetic outputs are visualized in real time, showing incremental improvements across loops - such as enhanced facial structure, thermal texture realism, and pose diversity.

**4. Loss Monitoring and Validation**

**Fig. 8:**
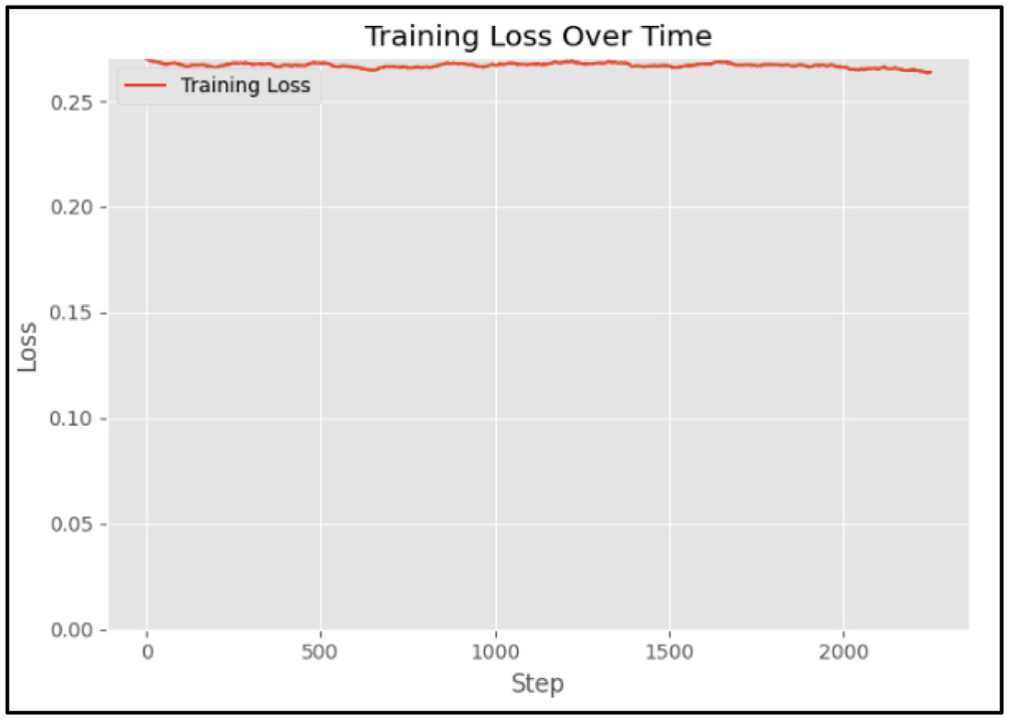
Training accuracy graph: the training loss remains stable, indicating consistent convergence during FLUX thermal-domain fine-tuning on the GPU-cluster setup using ComfyUI.

Each training loop connects to a VisualizeLoss node that plots real-time loss metrics. Loss curves are displayed for each iteration as shown in Fig. 8, indicating stable convergence and reduced reconstruction error over time. Validation steps are executed through FluxTrainValidate nodes using a subset of held-out images to ensure the model generalizes across different thermal conditions.

Unlike discriminative models, diffusion models are optimized using a noise-prediction (or velocity-prediction) objective, where the model learns to predict noise added at randomly sampled diffusion timesteps. As a result:

• The absolute loss magnitude is not directly correlated with perceptual sample quality
• The loss typically stabilizes early and fluctuates within a narrow band
• Large monotonic decreases, as seen in classification or regression tasks, are not expected

In our case, the relatively flat and stable loss curve indicates that the model has converged to a steady noise-estimation regime, rather than suffering from optimization instability or underfitting. This behaviour is consistent with prior diffusion-based works, where training progress is primarily assessed via qualitative sample quality and downstream task performance, rather than loss decay alone.

Moreover, during fine-tuning from a pretrained FLUX diffusion checkpoint, the model begins in an already optimized region of the parameter space. Consequently, only minor loss adjustments are required to adapt the model to the thermal facial domain, which further explains the limited loss variation observed during training.

**Fig. 9:**
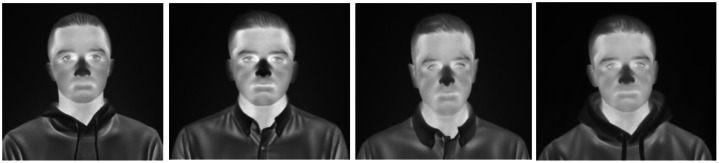
Thermal facial samples generated at different stages of fine-tuning of the FLUX diffusion model.

To better reflect training dynamics, we emphasize that sample fidelity, identity preservation, and thermal realism, which are the primary indicators of successful diffusion model fine-tuning. Crucially, to ensure that convergence is not judged from loss alone, we provide generated samples from multiple fine-tuning stages as shown in Fig. 9, demonstrating progressive improvements in:

• thermal realism
• identity preservation
• pose and expression consistency.

This is consistent with established diffusion-model practice, where sample quality and semantic fidelity are the primary indicators of training progress.

## 5 Model saving and output generation

Once training stabilizes, the model weights are saved periodically through FluxTrainSave, producing LoRA checkpoints. The final fine-tuned model generates 512 × 512 2D thermal facial frames using the inference script as shown in below code snippet, representing facial expressions, head poses, and accessory variations. The output visualization node as depicted in rightmost section of the Fig. 3 displays final synthesis results - showing distinct identities, gender balance, and pose variation consistency.



***Stage 2: Image-to-Video Translation (S2)***

**Fig. 10:**
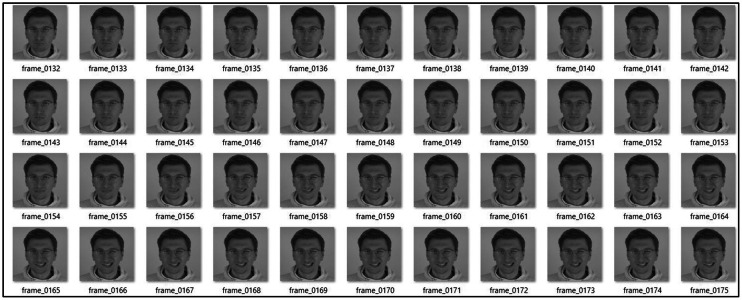
Image data samples extracted from two distinct sets of driving videos. The first set (D1) depicts head pose variations whereas second set (D2) depicts facial expressions.



Following the generation of 2D thermal facial frames using the fine-tuned Flux-LoRA diffusion model, a video retargeting and animation framework was developed to synthesize realistic thermal face motion sequences. This stage integrates an image-to-video translation pipeline with enhanced stitching and retargeting control to ensure natural temporal coherence and stable identity preservation across frames. The images generated in S1 were animated by loading the driving videos (D-1, D-2) as shown in below code snippet. The frames from both the driving videos sets are shown in Fig. 10, providing motion references for facial expressions and head-pose transformations.

**1. Backbone Model**

We adopted the LivePortrait framework [4] as the base module for this purpose, due to its robust canonical motion mapping and pose generalization capabilities. Within this system, the model M unifies three submodules - the canonical keypoint detector L, head pose estimator H, and expression deformation predictor Delta Δ - using a ConvNeXt-V2-Tiny backbone [12].

**2. Thermal Domain Adaptation**

A custom thermal preprocessing step was implemented to normalize pixel intensity distributions and preserve spatial gradients while mitigating domain noise as shown in below code snippet. This preprocessing enhanced the consistency of keypoint detection and motion tracking under varying temperature contrasts. Additionally, specific layers of the LivePortrait model [4] were selectively fine-tuned to adapt to spectral differences between visible and infrared modalities, emphasizing shape and motion continuity rather than fine RGB-textural details.



**3. Facial Retargeting and SPADE-ResNet Integration**

**Fig. 11:**
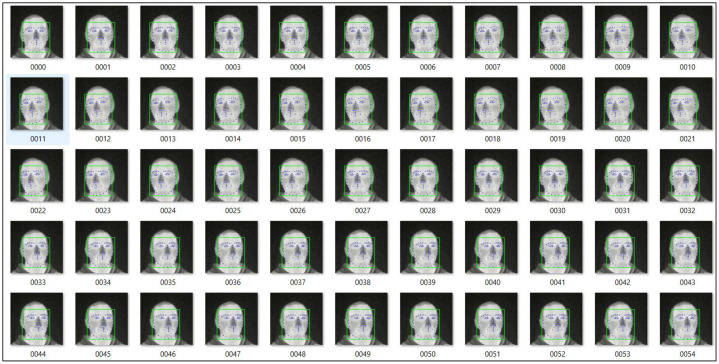
Facial landmarks extracted using thermally tuned landmark detector [11,10] for finetuning canonical keypoint detector L.

To enhance facial dynamics and realism, the pipeline integrates stitching, eye retargeting, and lip retargeting modules fine-tuned for thermal imagery. Thermal faces often exhibit distinct contrast gradients in the eyes, lips, and contour regions; therefore, the canonical keypoint detector L was refined using a thermally tuned landmark detector [13,14], ensuring accurate localization of thermal-invariant landmarks across subjects as shown in Fig. 11.

The Spatially Adaptive Normalization (SPADE) decoder G was incorporated within a ResNet framework to effectively reconstruct and animate thermal sequences as shown in below code snippet. This decoder interprets warped thermal feature volumes fs as semantic maps, preserving both structural detail and smooth motion. These modules preserved frame-to-frame consistency in the LWIR domain, ensuring smooth temporal transitions and maintaining the underlying thermal distribution across frames.



***Tools and Implementation***

• **Software Frameworks:** ComfyUI (for pipeline orchestration), Python (for preprocessing, image to video translation and annotations).
• **Models:** FLUX.1 DEV UNET, VAE, CLIP (text encoder), Flux LoRA module.
• **Hardware:** Quad NVIDIA A6000 GPU (≥ 48 GB VRAM) Server Machine.
• **Generation Parameters:** Step size = 22–32; CFG scale = 3; Dual CLIP conditioning with positive/negative prompts.
• **Output Format:** 512 × 512 resolution, stored as MP4 (video) and PNG (frame) files with corresponding face detection annotations (TXT).

***Algorithm: ThermVision-DB Image Generation and Video Retargeting Pipeline***

The complete pseudo code for the ThermVision data generation and animation workflow, outlining both the S1: Thermal Face Generation phase (using the fine-tuned Flux-LoRA diffusion model) and the S2: Image-to-Video Retargeting phase (using LivePortrait with thermal domain adaptation) and provided below. Both the algorithm summarizes the key computational steps, including dataset initialization, model training, prompt conditioning, and motion-based video synthesis for generating LWIR thermal facial sequences.

*Phase S1: Thermal Face Generation (Flux-LoRA Diffusion)*



*Phase S2: Video Retargeting and Animation (LivePortrait Thermal Adaptation)*



DATASET APPICABLE SCENARIOS

The proposed thermal face dataset supports a wide range of applications, including:

1. ***Thermal face recognition*** for reliable identity verification in low-light, nighttime, and illumination-invariant environments.
2. ***Privacy-preserving biometric systems*** where thermal imagery inherently limits the capture of identifiable visible facial details.
3. ***Driver Monitoring Systems (DMS)*** including driver identification, face tracking, and attention monitoring under challenging lighting conditions.
4. ***Surveillance and access control*** in security-sensitive and restricted environments.
5. ***Human presence and face detection*** in adverse environmental conditions such as fog, smoke, or low visibility.
6. ***Training and evaluation of generative models***, including diffusion-based thermal face synthesis and data augmentation.
7. ***Robustness assessment*** across pose, distance, and temperature variations for real-world thermal vision deployment.

## Limitations

Through this work and by releasing the ThermVision-DB dataset we aim to provides a diverse and high-quality collection of synthetic LWIR facial data, however there are certain limitations that should be acknowledged.

1. ***Lack of Paired RGB-Thermal Facial Data:*** The dataset is restricted to the thermal domain and does not include paired RGB-LWIR facial images. Although such paired data would be valuable for multi-modal and cross-domain research, identity-consistent RGB synthesis from LWIR facial imagery remains technically infeasible. Diffusion-based cross-domain generation requires large-scale, identity-aligned RGB-LWIR training data, which are currently unavailable. Moreover, fine-grained identity cues essential for realistic RGB face synthesis, such as skin tone, pigmentation, facial hair, and micro-textural details are largely absent or severely attenuated in LWIR imagery. Consequently, generating RGB faces from thermal inputs would necessitate hallucination of identity-critical features, rendering the results unsuitable for biometric or identity-preserving research.
2. ***Thermal-Specific Model Scope:*** The FLUX diffusion model employed in this work is exclusively fine-tuned on LWIR thermal facial data, enabling accurate modelling of thermal-specific characteristics such as heat distribution patterns and sensor-dependent noise. Extending this framework to RGB image generation would require a fundamentally different training paradigm, involving paired multi-modal supervision or physics-aware rendering pipelines, which are beyond the scope of the present study.
3. ***Residual Biases in Attribute Realism:*** Despite the use of prompt engineering and dual CLIP conditioning to promote balanced gender and facial attribute representation, minor biases may persist in texture quality and expression realism. These artifacts stem from uneven representation and latent biases inherited from the source data used during generative model training*.*
4. ***Occasional Generative Artifacts Under Extreme Conditions****:* While the thermally tuned LoRA model produces robust results in most scenarios, occasional artifacts may arise under complex poses or extreme yaw angles. These include thermal noise hallucinations, unrealistic temperature distributions, and partial degradation of facial structure, particularly in challenging geometric configurations.

*Overall, these limitations reflect current technical constraints in thermal image synthesis and cross-modal generation. Addressing them represents promising directions for future research, particularly as larger paired datasets and more advanced cross-modal diffusion methods become available.*

## Ethics Statement

The authors have read and follow the ethical requirements for publication in Data in Brief and confirming that the current work does not involve human subjects, animal experiments, or any data collected from social media platforms.

## Credit Author Statement

*Please outline the contributions of each co-author, using the categories listed on this webpage.*

**Muhammad Ali Farooq:** Conceptualization; Methodology; Software; Data curation; Formal analysis; Investigation; Visualization; Validation; Writing – original draft; Writing – review & editing; Project administration; **Waseem Shariff:** Software; Validation; Writing – review & editing; **Peter Corcoran:** Supervision; Conceptualization; Funding acquisition; Resources; Validation; Writing – review & editing; Project administration; Resources.

## Data Availability

Hugging FaceThermVision-DB (Original data).
